# The Influence of Nutritional and Lifestyle Factors on Glioma Incidence

**DOI:** 10.3390/nu12061812

**Published:** 2020-06-17

**Authors:** Joanna Bielecka, Renata Markiewicz-Żukowska

**Affiliations:** Department of Bromatology, Medical University of Białystok, 15-221 Białystok, Poland; renmar@poczta.onet.pl

**Keywords:** nutritional factors, glioma, prevention

## Abstract

Cancers are the first main cause of premature death in developed countries. Since brain tumors, especially gliomas, are the most lethal type of cancers, risk factors for their prevalence are still being discussed. Nearly 30–50% of all cancers could be prevented by proper nutritional habits and other lifestyle factors, but their influence on the tumors of the central nervous system has not been explained completely and still requires further studies. That is why we attempted to review the available research in this field, with a special focus on the factors with the proven protective activity observed in other cancers. Adequate vegetables and antioxidants (such as vitamins C and A) provided with a diet could have a protective effect, while other factors have shown no correlation with the incidence of glioma. However, further studies are necessary to determine whether fish, coffee, and tea consumption may prevent glioma. Maintaining proper body weight and undertaking a sufficient level of daily physical activity also seem to be important. Excessive body mass index (BMI) and higher attained height have increased the risk of glioma. In order to link more accurately the chosen factors to the prevalence of gliomas, it seems necessary to conduct large cohort, prospective, controlled studies in different world regions.

## 1. Introduction

Cancers are the first main cause of premature death in developed countries; this has been confirmed in 48 (28%) of 172 study countries among people under the age of 70, based on the epidemiological data of the World Health Organization (WHO). According to global cancer statistics assumptions in 2018, there were reported 18.1 million new cancer diagnoses and 9.6 million cancer deaths. Lung cancer and breast cancer were diagnosed most frequently—each one constituted 11.6% of new cases. The highest mortality of all cancer deaths was caused by lung cancer (18.4%). Regarding the available statistics for both sexes, brain tumors are not included in the 10 most common cancers and in the 10 cancers that cause the highest number of deaths. Despite this fact, the comparison of the age-standardized rate data for tumors incidence of the brain and the central nervous system (CNS) (3.9/100,000 in men and 3.1/100,000 in women) and mortality (3.2/100,000 in men and 2.3/100,000 in women) indicates extremely poor prognosis for this group of patients [1].

However, in the group of brain tumors, in particular, malignant gliomas are the most lethal type of cancer. In 2019, a total of 296,851 new cases of cancers of the brain and the central nervous system were diagnosed, of which 52.5% were confirmed in Asia, 21.8% in Europe, 10% in Latin America and Caribbean, 9.1% in North America, 5.7% in Africa, and 0.82% in Oceania. The cumulative risk (CR) for the incidence of these types of cancers is the highest for Southern Europe and has been calculated as 0.65% and is the lowest for Western Africa, i.e., 0.11%. The CR in Australia and New Zealand has been established to be 0.56%, in North America as 0.52%, and 0.48% in South America, whereas, in Asia regions, the greatest CR has been determined in the Western Asia—0.49%. The CR of very high human development index countries (HDI) is higher than the CR of low HDI countries (0.58% and 0.13%, respectively) [1]. In countries with high HDI, the western lifestyle is more often reported. This lifestyle includes highly processed, convenience food along with fast food consumption, sugary soft drinks intake, which results in the deficiency of vitamins, minerals, and dietary fiber. Following the western diet for a long time with a combination of a sedentary lifestyle may result in the weight gain, pathological changes in lipids, and energy metabolism, as well as activation of the immune system. Chronic metabolic inflammation contributes to the development of non-communicable diseases (NCD), among which certain cancers are also listed—colon, pancreatic, and breast cancer [2].

Gliomas are often diagnosed in the advanced stage and are poorly responsive to currently available treatment. Glioma is the most prevalent type of CNS tumor in adults and constitutes 70% of all brain tumors [3]. Based solely on the histological diagnosis, gliomas originate from astrocytes and oligodendrocytes. Currently, with accordance to the new WHO classification of gliomas that integrates molecular alterations and histology, five glioma subtypes are defined. Regarding their degree of malignancy, I and II are low-grade, and III and IV are high-grade gliomas (HGG). Tumors classified as grade I grow slowly, are benign, and have a good prognosis for long-term survival. Grade II tumors also tend to develop slowly, but they can recur as more malignant tumors. HGG is always malignant, and grade III tumors may progress to a higher grade of glioma, but this is dependent on their isocitrate dehydrogenase (IDH) mutational status. IDH mutants progress to GBM (*glioblastoma multiforme*) and are considered secondary GBMs. Primary GBMs develop *de novo* and are molecularly distinct from secondary GBM. GBMs are the most common astrocytic glioma and are highly aggressive and lethal [4]. Fast-growing and very malignant tumors are classified as grade IV. GBM is very heterogeneous in nature, especially in terms of its histological subtypes [5]. The median survival of patients with the most aggressive form of GBM varies between 10 to 15 months despite the therapy [3].

The current treatment regimen varies depending on the tissue diagnosis and the selected molecular markers assessment. Younger age and better performance status are considered as therapy-independent prognostic factors. Whenever feasible, neurosurgical removal of the tumor with a safety margin for healthy tissue to improve neurological function is recommended in all newly diagnosed patients. The schedule of radiotherapy is also determined with regard to diagnosis and aims to improve the local control and preserve the patient’s function. Additionally, chemotherapy is included in the standard care for most glioma patients. The most commonly used cytotoxic agents are temozolomide, procarbazine, and lomustine. Due to difficulties associated with complete resection of glioma, the actions taken to prevent its progression play an important role in the treatment of this type of brain tumor [6]. 

To date, 25 risk factor loci and several rare inherited mutations that may cause glioma in some families have been identified, but ionizing radiation is the only confirmed environmental risk factor [4]. It is estimated that 30–50% of all cancers could be prevented by modifications in lifestyle, of which smoking, alcohol consumption, dietary patterns, a level of physical activity, and the weight of the body have been considered in the available literature. Several dietary habits are well established as risk factors for many human cancers. For example, in 2015, the IARC (International Agency for Research of Cancer) classified consumption of processed meat as cancerogenic to humans (group 1 of carcinogens) for colorectal cancer. Moreover, alcohol intake at the level of 30 g per day or more is also described as a risk factor for the incidence of this type of cancer. Foods preserved by salting could enhance stomach cancer development [7]. On the other hand, many bioactive compounds have been described as supporting a cancer-preventing process. Whole grains and foods containing dietary fiber and phytochemicals could have a protective effect contrary to colorectal cancer incidence. Non-starchy vegetables or fruit probably protect against several cancers of the aerodigestive tract. Furthermore, coffee consumption (both caffeinated and decaffeinated) could decrease the risk of endometrial cancer [7]. The influence of a diet on the central nervous system (CNS) tumors, especially glioma, has not been determined sufficiently. The currently available literature provides a meta-analysis as well as results of cohort studies, describing the relationship between selected dietary factors (tea and coffee; fruits and vegetables; fish, meat, and N-nitroso compounds; vitamins A, C, E) and the risk of developing glioma. That is why we attempted to review the research available in this area to systematize and sum up the information on this topic. The article focused on the importance of nutritional and lifestyle factors in the development of gliomas among people and indicated what might play a significant role in reducing the risk of glioma incidence.

## 2. Material and Methods

This paper was based on the articles published in English-language journals until December 2019, available in three electronic databases: Scopus, PubMed, and Google Scholar, which meet required quality standards in relation to the information on the dietary and lifestyle factors discussed in this paper and their association with the prevalence of gliomas. The articles were searched by using the following keywords: “glioma”; “glioblastoma”; “brain tumor” in collocations with “diet”; “nutrition”; “lifestyle factors”; “alcohol”; “tea”; “coffee”; “antioxidants”; “vitamin A”; “vitamin C”; “vitamin E”; “fruits”; “vegetables”; “meat”; “red meat”; “n-nitroso compounds”; “fish”; “fatty acids”; “physical activity”; “body weight”. We adopted the following inclusion criteria: the research in the study topic, specifying glioma cases, with the full text available. The individual studies incorporated in the meta-analysis on the selected topic were excluded from the overall review.

## 3. Results and Discussion

### 3.1. Nutritional Factors

#### 3.1.1. Alcohol Consumption

Alcohol consumption is one of the strongest lifestyle-related carcinogens. It is considered to be a risk factor for brain tumors because of its ability to cross the blood-brain barrier and a neurotoxic effect. This may lead to structural changes in the hippocampus in adolescence and decreased brain volume (white and grey matter) in adulthood, probably contributing to the changes in the axonal integrity and myelination [8]. The pro-cancerogenic alcohol effect has already been confirmed in several cancers. In accordance with the position of IARC (International Agency for Research of Cancer) based on the publication of Congliano et al., there is sufficient evidence among the study population to indicate alcohol consumption as a modifiable risk factor in the development of the following cancers: oral cavity, pharynx, esophagus, colon and rectum, liver and bile duct, larynx, and breast (in females) [9].

The negative impact of alcohol consumption has been demonstrated not only in the pathogenesis of selected tumors but also in cancer survivors. In the large meta-analysis study, focusing on the assessment of dietary factors in the patients cured of cancer, drinking alcohol was positively linked to the overall mortality (relative risk (RR): 1.08, 95% CI: 1.02–1.16) [10]. Nevertheless, the research results on the impact of alcohol consumption on glioma risk have not been conclusive. Qi et al. [11] performed a meta-analysis of 19 observational studies (15 case-control and four cohort studies) to link the alcohol consumption with the risk of glioblastoma. There was no correlation found here as the combined RR was 0.96 (95% CI: 0.89–1.04). Considering the geographical origin in European, Australian, and Asian studies incorporated in the meta-analysis by Qi et al., no important correlations were found, either. Interestingly, North American studies showed a significant association between alcohol consumption and the elevated glioma risk (RR: 0.78, 95% CI: 0.65–0.93) [11]. In the case-control study, Allès et al. [12] looked for the relationship between alcohol consumption and CNS tumors. This research was a part of CERENAT study (CEREbral tumors: a NATional study), which was a multicenter study trying to define the role of environmental factors contributing to the incidence of primary CNS tumors in adults. In this investigation, 1479 people were examined. The study group consisted of 494 cancer cases, of which 201 were neuroepithelial tumors, 193 meningiomas, 100 other CNS tumors, and the rest was a control group. The highest percentages of neuroepithelial tumors were glioblastomas (41.6%), followed by oligodendrogliomas (14.9%), oligoastrocytomas (12.9%), gliomas (9.4%), astrocytomas (7.9%), ependymomas (5.4%). Based on the statistical analysis, all neuroepithelial tumors were taken into account, without dividing them into individual types of neuroepithelial tumors. The data on alcohol consumption patterns were collected using standardized questionnaires. No significant correlation was observed between alcohol consumption and neuroepithelial tumors risk, but there was a linear decreasing dose-response relationship trend for meningioma (odds ratio (OR): 0.33; *p* = 0.001) and overall CNS tumors (including all studied groups, *p* = 0.003) and alcohol intake [12]. The investigation carried out by Kuan et al. [13] on three large prospective studies, taking into consideration the data divided into three time periods of observation (all follow-up, first 5 years of follow-up, and 5+ years of follow-up), showed no influence of alcohol consumption (counted per 10 g of alcohol) on the risk of glioma incidence [13].

Despite the fact that alcohol is an important risk factor, increasing the risk of developing certain types of cancer, based on the results above, alcohol consumption does not seem to enhance glioma risk significantly, but further investigations are required to confirm these conclusions.

The mechanisms by which alcohol could induce cancerogenesis is still not clear; nonetheless, the cellular stress, the genotoxic effect of acetaldehyde, inflammation, and altered folate metabolism are considered [14]. However, it should be mentioned that depending on the type of cancer, the effect of alcohol consumption is different—may have no influence or any pro-cancerogenic effect.

#### 3.1.2. Tea and Coffee

Coffee, a rich source of many health-promoting compounds, is the drink consumed most frequently in the world. However, it also contains dangerous substances, which are produced in the roasting process (e.g., acrylamide). Roasted coffee contains more than 1000 biologically active compounds, part of which could be beneficial for humans, characterized by the antioxidant, antifibrotic, antimutagenic, and anticancer activity [15]. Caffeine, chlorogenic acid, polyphenols, and also diterpenes like kahweol and cafestol belong to the most important compounds. The mechanisms that have the chemopreventive action include inhibition of the harmful effect that causes oxidative stress, repair of DNA damage, apoptosis, reduction of inflammation, and the influence on phase II enzymatic activity. Another mechanism considered as anti-cancerogenic is the inhibitory effect of kahweol on DNA methyltransferase and reactive methylation silenced genes [16]. Overall, coffee consumption decreases the risk of cancer development by 18% [15].

Tea is another popular drink that is often consumed. Its production involves processing *Camellia sinensis* leaves. Three basic types of tea are distinguished (green, black, oolong), depending on the process carried out. Green tea, unlike black and oolong, is not fermented. Tea is a rich source of polyphenols (including catechins). However, special attention should be given to green tea catechins amid which epigallocatechin-3-gallate (EGCG) is the most common, while epicatechin-3-gallate (ECG), epigallocatechin (EGC), and epicatechin (EC) are found in smaller quantities. Many mechanisms of green tea catechins have been described, which incorporate anti- and pro-oxidant activity, apoptosis induction, transcriptional factors inhibition, influence on the immune system, epigenetic alternation, inhibition of the receptor tyrosine kinase pathways, and anti-metabolic syndrome effects [17]. EGCG may be used as an adjuvant in the therapy of glioma with temozolomide, irradiation, or cisplatin but not in bortezomib. Tests of EGCG in several glioma cells line have shown numerous potential beneficial mechanisms of the action, like limiting invasion, cell apoptosis, and preventing proliferation [18].

In 2013, Malerba et al. [19] conducted the meta-analysis on the relationship between tea and coffee consumption and glioma risk in adults. They included six papers, of which four were cohort and two case-control studies, and overall, there were 2075 glioma cases. All six studies provided information on coffee consumption, while tea consumption was included in four studies, and coffee combined with tea consumption in three studies. Hence, it should be noted that few studies were analyzed in this research. No relationship was observed between total coffee consumption and the risk of glioma for coffee drinkers and non-drinkers (or occasional drinkers) (RR: 0.96, 95% CI: 0.81–1.13). After taking into account the amount of coffee drunk (high vs. low consumption), no relevance was also observed (overall RR: 1.01, 95% CI: 0.83–1.22). Increasing daily consumption by 1 cup of coffee a day also had no effect (RR: 1.00, 95% CI: 0.96–1.05). The analysis of three studies on tea consumption showed a slight decrease in glioma risk in drinkers in comparison with non-drinkers (RR: 0.86, 95% CI: 0.78–0.94), but it was not confirmed after taking into account the amount of tea consumed (high vs. low, RR: 0.88, 95% CI: 0.69–1.12). The risk of glioma for people declaring the simultaneous consumption of larger amounts of coffee and tea seems to be lower compared to those drinking smaller amounts (RR: 0.75, 95% CI: 0.54–1.05) [19]. In 2018, Song et al. [20] conducted a meta-analysis that also included the research, followed by the publication of the work of Malerba et al. [19]. They examined 11 studies (eight were cohorts, and three were case-control studies), of which ten works concerned glioma and one the brain cancer (without division into subtypes). Among the studies providing the data about coffee consumption, the statistical analyses demonstrated that high coffee consumption might decrease glioma risk compared to its low intake (RR: 0.76, 95% CI: 0.55–0.97). Importantly, this protective effect was statistically significant. Seven studies contained information on tea consumption and the risk of developing glioma. However, the statistical analysis made by Song et al. [20] demonstrated no significant correlation between the factors mentioned above (RR: 0.85, 95% CI: 0.68–1.05) [20]. In the research of three large prospective studies from the United Kingdom (UK) and the United States of America (USA), Kuan et al. [13] specified three time-subcategories during the follow-up time—all follow-up, 5 years of follow-up, and 5+ years of follow-up—taking into consideration a possible impact of preclinical disease on dietary intake in cancer patients. In the case of coffee and tea consumption, no effect on glioma was observed, taking into account both the entire observation period and after excluding the first 5 years of follow-up [13]. In 2019, the combined data from the Nurses’ Health Study (I and II) and the Health Professionals Follow-Up Study (HPFS) were studied by Cote et al. [21]. Consumption of tea and coffee was assessed based on the information provided in the validated food frequency questionnaires. Glioma was diagnosed in 554 participants and confirmed in the medical records. Neither in females, nor in males, a relevant association was observed between caffeinated, decaffeinated, and total coffee intake and risk of glioma development. Interestingly, an inversely borderline significant association was detected for tea consumption higher than 1 cup/week (hazard ratio (HR): 0.73, 95% CI: 0.49–1.10). Though when the gender breakdown was included, the relationships were not confirmed. Moreover, a relevant inverse relationship was determined between tea consumption at the baseline and glioma risk in the pooled analysis (*p* = 0.02) and in females (*p* = 0.05) but not in males (*p* = 0.22) [21].

The individual analysis indicated no unequivocal relationship between the consumption of coffee, tea, or combined drink of both beverages and risk of glioma. According to the data above, none of the studies has found that tea and coffee consumption increases the risk of glioma, but more research is required to prove whether they truly can prevent this type of cancer. As mentioned at the outset, coffee and tea ingredients generally have an anti-cancer effect. However, there are only a few studies regarding the anti-glioma activity. In the laboratory study on the mouse xenograft model, caffeine, which is the central nervous system stimulant, reduced the tumor mass and prolonged survival. In the in vitro model, it was demonstrated that caffeine inhibited the Ca^2+^ release channel— inositol 1,4,5-trisphosphate receptor (IP3R) subtype 3—and, consequently, slowed glioblastoma invasion and migration [22].

Further studies are necessary to prove and explain the mechanisms of the inhibitory influence of bioactive compounds on glioblastoma development.

#### 3.1.3. Fruits and Vegetables

High intake of fruits and vegetables (their consumption recommendations range from 400 g per day, according to the WHO, to 640–800 g per day, in the USA) is one of the elements of a healthy lifestyle suggested to reduce the risk of cancer incidence. In the meta-analysis, taking into consideration the random relations, Aune et al. [23] observed, if the associations were causal, that in 2013, nearly 5.6–7.8 million premature deaths could have been a result of insufficient intake of fruits and vegetables (below 500 g and 800 g per day). They also observed that the consumption of 500 g fruits and vegetables could be beneficial in preventing overall cancer development. In addition, in the subgroup analysis, they found an inverse association between green–yellow and cruciferous vegetables and the total cancer risk [23].

Ying [24] conducted the meta-analysis based on 15 articles, of which 13 were case-control and two cohort studies, checking the relationship between the intake of fruits and vegetables and glioma risk. This study included the research that described the consumption of vegetables and fruits in the total count, without subdivision into subgroups (e.g., citrus and other fruit). The studies that recorded the consumption of fresh fruit or vegetables were also included. In individual studies, consumption levels were categorized by tertiles, quartiles, or quintiles, resulting in the division into a high and low intake group. In the research carried out by Holick et al. [25], which was incorporated in the meta-analysis by Ying [24], the fruits and vegetables intake levels were determined by quintiles for portions. One portion of vegetables was specified, e.g., as an equivalent of a half cup of broccoli, and, for fruits, it was one banana. The reported fruit consumption ranged from 0.8 to 4.1 portions per day, and for vegetables from 1.2 to 5.0 [25]. Exact intakes were not described in Ying’s meta-analysis. The data on the correlation between the consumption of vegetables and the risk of developing glioblastoma included a group of 5562 glioma cases from 15 studies (14 case-control studies and one prospective study) [24]. In seven of these studies, the inverse relationship between vegetable consumption and glioma risk was found, while the rest of the studies showed no significant relationship between these factors. In the overall summary of all studies, Ying established the inverse association between vegetable intake and the risk of glioma. It was shown that high compared to low vegetable intake was less related to the risk of glioma in the overall analysis (RR: 0.78, 95% CI: 0.69–0.87). However, in the ethnic specified groups, the results were relevant, solely among Asians (RR: 0.79, 95% CI: 0.68–0.91) and not in white (RR: 0.78, 95% CI: 0.65–0.95) [24]. In order to assess the correlation between fruit consumption and the risk of glioblastoma, the results of 17 studies from 15 articles, including 3994 glioma cases, were selected (15 case-control studies and two prospective studies). In more than half (in 10 of 17) of the studies analyzed, there was no significant relationship between the fruit consumption and the glioblastoma risk, while only the remaining seven studies reported a reduction in the risk of glioblastoma. However, Ying [24] finally concluded that there was no significant association between fruit consumption and the glioblastoma risk (RR: 0.83, 95% CI: 0.66–1.04). Nevertheless, the analysis of an ethnic group showed that fruit consumption might have a protective effect on glioblastoma, solely among Asians (RR: 0.57, 95% CI: 0.35–0.95). In the CERENAT case-control study, neither a vegetable nor fruit consumption was associated with an effect on the development of neuroepithelial tumors (including glioma and GBM) [12]. Weak evidence on the increasing risk of glioma in relation to higher total fruit (per 100 g), citrus fruit (per 100 g), and fiber (per 5 g) consumption standardized to 1600 calories (kcal)/day in women and to 2000 kcal/day in men was found in the research of Kuan et al. [13]. After excluding the first 5-years of follow-up, no such dependencies were observed [13]. The significant reverse association between the intake of yellow-orange and the green leafy vegetables and the possibility of glioma incidence was found in the multicenter study conducted by Terry et al. [26]. The data collected from 1548 brain cancer cases were taken into consideration, of which 1185 were glioma cases. The levels of individual food group consumption were divided into quartiles of the intake reported. In their investigation, higher compared to low intake of these vegetables was related to the reduced glioma risk (OR: 0.7, 95% CI: 0.5–0.9, *p* < 0.001). In the analysis of individual tumor histology subtypes, the researchers determined that these associations were strongest in astrocytomas and glioblastomas [26].

Protection and repair of DNA damage, modulation of DNA methylation, induction of phase II detoxifying enzymes, and promotion of apoptosis are mentioned among the mechanisms responsible for the protective effect of eating fruits and vegetables. The above mechanisms of fruits and vegetables action are attributed to the presence of numerous anti-tumor compounds, such as vitamins, flavonoids, isothiocyanates, glucosinolates, and dithiolthiones [27].

Although the protective effect of eating vegetables has not been demonstrated in all individual studies, the results of the meta-analysis carried out by Ying deserve attention. A sufficiently high intake of vegetables could be an element of glioma prevention, especially among Asians. In turn, the beneficial influence of fruits intake has not been sufficiently documented for this type of cancer. However, it is vital to consume an appropriate amount of fruits because of their preventive role in reducing the incidence of other types of cancers.

#### 3.1.4. Fish Intake

Eicosapentaenoic acid (EPA) and docosahexaenoic acid (DHA), included in polyunsaturated fatty acids n-3 (PUFAs), have numerous positive effects on the human body function. PUFAs should be provided with a diet because the human body can biosynthesize them only in small amounts. The dietary source of EPA and DHA is seafood [28].

Due to protective EPA and DHA effects on brain tissues, it can be suggested that fish intake could also reduce glioma incidence. In the meta-analysis, Lian et al. [29] looked for a link between fish consumption and the risk of developing brain tumors in nine observational studies, but gliomas were listed only in five of them. All these studies, including 501,617 participants, of which 4428 brain cancer cases were diagnosed, were carried out and published between 1986 and 2006. No significant relationship was established between fish intake and glioma risk (RR: 0.81, 95% CI: 0.64–1.03). However, the results of the meta-analysis showed that higher fish intake was significantly inversely associated with the risk of brain tumors (RR high compared to low consumption: 0.83, 95% CI: 0.7–0.99). Additionally, an increase in fish consumption by 100 g/week was also related to the reduced risk of brain cancer incidence [29]. Similar results were obtained in the combined research of three large prospective studies from the USA and the UK based on the data collected from over 1 million participants (*n* = 1,262,104, glioma cases: 2313). Fish consumption had no effect on glioma risk, both in overall follow-up and after excluding the first 5 years of follow-up [13]. In the case-control study carried out by Dadfarma et al. [30], the data from 128 newly diagnosed Iranian adult glioma patients and 258 controls were analyzed based on the food frequency questionnaire. The authors checked the relations between dietary PUFA intake categorized by quartile cut-points and glioma incidence. In this study group, higher PUFA (highest quartile) intake was less significantly associated with glioma incidence than its lower consumption. This relation was found after considering age, sex, and total energy intake [30].

The balance between PUFA n-3 and PUFA n-6 seems to play a crucial role in the formation of pro- and anti-inflammatory lipids. Recently, attention has been paid to pro-cancerogenic effects of omega-3 fatty acids, but this relationship could exist solely in individuals with specific genetic or metabolic predispositions. It is unclear whether the pro-cancerogenic effect is caused by pollution found in the marine food or by the imbalanced proportion of n-3:n-6 fatty acids in a daily diet [31].

Having reviewed the currently available literature, it seems that fish consumption has no significant impact in the case of glioma development, but the protective effect of fish consumption in other brain cancer suggests a possible protective activity against cancerogenesis in the brain tissues. However, further research should be carried out to explain this mechanism.

#### 3.1.5. Red Meat and N-Nitroso Compounds

According to IARC, processed meat has been classified as cancerogenic to humans, and this effect refers to colon and rectum cancer. Red meat consumption has been determined as probably cancerogenic to humans, especially regarding colon, rectum, pancreas, and prostate cancer [9]. The cancerogenic effect of red processed and red meat consists of many mechanisms, among which N-nitroso compounds (NOC) formation and heme iron influence are considered the most significant. It is worth emphasizing that nitrate and nitrite are classified as cancerogenic agents with insufficient evidence in humans in case of stomach cancer [9]. The main sources of NOC exposure are processed meats, for example, bacon, sausages, and salami. However, the precursors of NOC-nitrate and nitrite may occur in drinking water and in vegetables. It is estimated that bacteria in the oral cavity could be responsible for nearly 5% of nitrate conversion. The next stage of the endogenous transformation of NOC precursors takes place in the stomach. It is assumed that a significant proportion of NOC exposure results from endogenous alternations and accounts for 45–75% of the exposure [32]. Meat processing also might result in the formation of heterocyclic amines (HCAs) and polycyclic aromatic hydrocarbons, which could induce cancerogenesis in the various organs, including the brain. These compounds have the ability to cross the brain-blood barrier. The main dietary sources of HCAs are cooked meat and fish. In animal models, HCAs are confirmed to be cancerogenic. Human epidemiological studies have confirmed their cancerogenic effect for bladder, pancreas, and renal cell cancer [33].

A meta-analysis conducted by Wei et al. [34], taking into account the data from 14 articles, provided the information on processed and red meat consumption and glioma development risk in 877,640 participants and 3896 glioma cases. No specific consumption levels were established in this study, e.g., in terms of portions. Considering individual results of the research, positive relations between the processed meat intake and the glioma incidence were shown in two papers, and in the remaining 12 papers, there were no associations. However, the pooled results comparing the high with the low red processed meat consumption demonstrated a relevant association with glioma risk (RR: 1.25, 95% CI: 1.08–1.45). In the population subgroup analysis, these relations were statistically significant among the studies conducted in the USA (RR: 1.28, 95% CI: 1.09–1.50). Depending on the study design, the differences were also shown. Though, no statistically relevant association was demonstrated in the cohort studies (RR: 1.10, 95% CI: 0.88–1.37), but only in the case-control studies (RR: 1.33, 95% CI: 1.09–1.62). The analysis of the data obtained from 1156 people with glioma, included in the three studies, showed no significant association between the consumption of red meat and the risk of glioma development (RR: 0.89, 95% CI: 0.71–1.12) [34]. However, the meta-analysis of Saneei et al. [35], which included the data from six studies, showed that the unprocessed red meat intake was significantly related to the risk of glioma development (RR: 1.30, 95% CI: 1.08–1.58). The relations did not change when the two studies were excluded, in which fish and poultry were counted in the same category as red meat. The data from 17 papers were used to count the RR for the high compared to the low unprocessed meat intake and glioma development at 1.14 (95% CI: 0.98–1.33). Despite the lack of important relations, there was a little positive increasing trend between processed meat and the incidence of glioma. In the population-based studies, high processed meat intake was linked to a 26% higher glioma incidence, while no associations were shown in the cohort studies. On the other hand, in the hospital-based case-control studies, red meat consumption decreased the glioma risk by 21%. In turn, considering the total red meat consumption, it did not seem to increase the development of glioma (RR: 1.05, 95% CI: 0.89–1.25). The results did not differ much when the study reporting processed and unprocessed meat consumption in one group was excluded [35]. However, the authors did not indicate the approximate portions or levels of consumption in any of the studies performed.

Ward et al. [36] studied the data from the prospective cohort study (European Prospective Investigation into Cancer and Nutrition-EPIC study) based on the information collected from nine European countries, including 408,751 participants. The average follow-up time was 14.1 years, and there were diagnosed 688 glioma cases. Dietary assessment was conducted by using questionnaires specified for each country. No associations between the intake of red meat, processed meat, and glioma incidence were found. Red meat consumption was counted per 10 g/day and standardized to 1000 kcal; processed meat per 5 g/day and also standardized to 1000 kcal. Similarly, when intake of total iron and heme iron was specified, no relations were established. The intake of total iron was calculated at 6.33 mg/1000 kcal and for heme iron at 0.54 mg/1000 kcal. The relations did not change when the period of the first 2-years of follow-up was excluded because of possible modifications in dietary patterns in undiagnosed glioma cases [36].

In turn, no relations were shown between the NOC precursors, such as nitrate and nitrite intake, and the glioma risk in the meta-analysis by Wang et al. [37]. They investigated the data provided from nine studies incorporated in seven articles, of which two were cohort and seven case-control studies. The overall information from 2264 glioma cases was analyzed. Solely, one study reported a positive association between the nitrite intake and the glioma risk, while no associations were found in other studies. The high in comparison to low nitrite intake was not related to the risk of glioma development in adults (OR: 1.17, 95% CI: 0.99–1.35). The analysis results of nitrate intake and glioma development risk were similar, based on seven studies and 1771 glioma cases. Wang et al. [37] demonstrated that high compared to low nitrate intake was not associated with glioma risk (OR: 0.89, 95% CI: 0.66–1.12). No differences were found in glioma incidence, according to the study region. However, the study design might play a role in interpreting results because relations were significant in the case-control (OR: 0.72, 95% CI: 0.52–0.93) but not in the cohort studies (OR: 1.18, 95% CI: 0.87–1.47) [37]. The comparable results regarding the relationship between the dietary nitrate intake and the glioma risk were demonstrated in Xie et al.’s [38] meta-analysis. There were no relevant associations between the dietary nitrate intake and the adult glioma risk (RR: 1.02, 95% CI: 0.85–1.22). Though, referring to nitrite, it was demonstrated that the higher versus lower consumption was linked to the increased risk of glioma incidence (RR: 1.21, 95% CI: 1.03–1.42) [38]. In both studies—by Wang et al. and Xie et al.—no dose-response analysis was performed due to insufficient data and lack of the research that could compare the levels of high intake versus low.

Based on the available literature, it seems that iron intake (both heme and non-heme) has no influence on the glioma incidence risk. However, taking into account the results of meta-analyses, some studies seem to show a relationship between the consumption of red processed meat and the risk of glioma development, while other studies have shown no relationship. The correlation between the red unprocessed meat consumption and the incidence of glioma has not been proved explicitly.

#### 3.1.6. Antioxidants

Many compounds that could have a protective, delaying, or eliminating effect on oxidative damage in molecules are classified as antioxidants with different action mechanisms. Enzymes, such as catalase or glutathione peroxidase, and metal-binding proteins like ferritin and minerals (e.g., Zn, Se) are involved in the prevention of free radicals formation. Another group of antioxidative compounds is focused on preventing oxidative chain reactions by capturing free radicals. Such a mechanism of action is observed, among others, in the case of glutathione, vitamin E, vitamin C, and carotenoids, while the damage caused by free radicals is repaired by lipases, proteases, DNA repair enzymes, or similar compounds [39].

Vitamin E involves a group of compounds belonging to tocopherols (α, β, γ, and δ) and tocotrienols. Antioxidative mechanisms of vitamin E (primarily, the most biologically active of this vitamin—α-tocopherol) properties include the elimination of free radicals, induction of cell apoptosis, and an inhibitory effect on tumor growth and cancerogenesis [40]. Besides, vitamin E prevents the oxidation of polyunsaturated fatty acids and phospholipids. It is also possible that in the promotion phase, vitamin E affects the immune system. In addition, it has been shown that α-tocopherol could inhibit the formation of nitrosamines [41]. In Qin et al.’s [42] meta-analysis, vitamin E intake was not associated with the effect on glioma. The data from 12 studies, including 3180 glioma cases, were taken into consideration. Analyzing the collected studies individually, a decrease in the risk of glioma was reported only in two studies, while this relationship was not confirmed in the rest. A higher level of vitamin E intake was not significantly associated with the lower glioma risk compared to a lower level of intake (RR: 0.88, 95% CI: 0.69–1.12). After categorizing the data according to the study type (case-control *n* = 2260 RR: 0.85, 95% CI: 0.63–1.16; cohort *n* = 920 RR: 1.00, 95% CI: 0.77–1.13), no significant effect on the level of vitamin E intake was determined, either [42]. The data were insufficient to determine the dose-response relationship. In the research carried out by Dubrow et al. [43], which was incorporated in the meta-analysis by Qin et al. [42], median vitamin E intake levels ranged from 6.2 to 12.1 mg standardized per 1000 kcal. No effect of vitamin E, vitamin C, and β-carotene intake on the risk of glioma was observed in the combined data analyzed from three large cohorts (Million Women Study (MWS), the National Institutes of Health and the American Association of Retired Persons (NIH-AARP) Diet Health Study, and the Prostate, Lung, Colorectal and Ovarian (PLCO) Cancer Screening Trial). No changes in the dependencies were reported when the data from the period of the first 5-years of follow-up were excluded [13].

Vitamin A includes a group of compounds characterized by unsaturated hydrocarbons, in which retinol and its derivatives are included (like retinal and provitamins A-carotenoids). Among antioxidative mechanisms of vitamin A activity, preventing lipid peroxidation, regulating cell differentiation, and inhibition of cancer cell proliferation are also described, besides its participation in the induction of detoxifying enzymes. Specific binding proteins for the retinol and retinoic acid could work as hormones controlling cell differentiation and influence the transport of these compounds across the nuclear membrane. Regarding provitamins A, the β-carotene is most effectively converted to retinol, and its main property is to reduce free radicals and prevent their negative effects [41]. In the glioma cell models (line U87 and line SHG44), all-trans retinoic acid promoted apoptosis and showed an inhibitory effect on migration, invasion, and proliferation of these cells [44]. In another study, the strong inhibition of proliferation and migration in the primary cultures of human glioma cells was demonstrated as an effect of the action of natural retinoic acid forms (all-trans retinoic acid (RA), 9-cis-RA, 13-cis-RA) [45]. In the meta-analysis by Lv et al. [46], vitamin A was demonstrated as a compound, potentially lowering the risk of glioma. The authors of this research investigated the data on 1841 glioma cases and 4123 controls from seven articles with eight case-control studies. Although the individual results of the two studies showed that dietary intake of vitamin A might reduce the risk of glioma, the results of the vast majority of studies (five studies) showed no association between vitamin A and the glioma incidence risk. In addition, in one study, higher vitamin A intake was correlated with an increased risk of glioma. A summary of these studies in the meta-analysis showed that the higher vitamin A intake in comparison with the lower intake might be associated with a decrease in the risk of glioma (RR: 0.80, 95% CI: 0.62–0.98). Due to the insufficient information in the individual articles, it was not possible to assess the impact on the risk of developing glioma, depending on the level of vitamin A intake. Most of the studies analyzed by Lv et al. provided the data on high/low intake levels, but only a few authors added the information on the specific amounts of vitamin A intake (DeLorenze; Tertile 1 (T1): 257.7 µg; Tertile 3 (T3): 3757.6 µg) [46,47]. Furthermore, a significant relation was reported for vitamin A intake and a lower risk of glioma development in the American population (RR: 0.73, 95% CI: 0.59–0.91, *p* < 0.001). However, no similar dependencies were observed in other countries [46].

The next antioxidant vitamin, largely responsible for neutralizing free radicals, is vitamin C, which is most common in fruits and vegetables. One of the known anticancer mechanisms of vitamin C is the inhibition of nitrosamines formation by blocking the nitrosation reaction. Besides, vitamin C stimulates the immune system, participates in the protection against oxidative DNA damages, and inhibits the activation of carcinogenesis, especially, at the phase of tumor promotion [41]. It was demonstrated that the lipophilic derivative of vitamin C-ascorbyl stearate-had a proapoptotic and antiproliferative effect in T98G glioma cells. This probably resulted from the influence on the insulin-like growth factor-I receptor expression, which promoted cell apoptosis [48]. Among the American population, Zhou et al. [49] demonstrated the protective effect of vitamin C intake on reducing the risk of glioma. They conducted the meta-analysis of 15 studies (13 were case-control studies) with 3409 participants on the impact of vitamin C consumption and glioma risk. The authors could not categorize the results according to a specified level of vitamin C intake due to a lack of data in most articles. Only individual papers provided the information about detailed consumption—in Dubrow et al.’s [43] study, the consumption of vitamin C ranged from 4.5 mg (T1) to 149.6 mg (T3) standardized to 1000 kcal. The majority of the articles investigated were carried out in the USA (11 out of 15), and the remaining single studies were conducted in other regions. Hence, this could be the reason for the lack of linkage in the relationship studied regarding vitamin C consumption and the risk of developing glioma in other regions. Most of the research (13 studies) proved no important associations between the vitamin C intake and the risk of glioma, while the inverse relationship was shown only in the remaining two studies. The summary of all collected articles indicated that the higher vitamin C intake versus its lower intake was significantly related to the lower glioma risk (RR: 0.86, 95% CI: 0.75–0.99). Additionally, the researchers demonstrated that this relation was relevant solely in the case-control studies (RR: 0.80, 95% CI: 0.69–0.93) [49].

Zinc has also been proved to possess antioxidant properties. It takes part in protection against oxidative stress, DNA replication, and protein synthesis and is an important micronutrient for brain health. Zinc is a crucial element for human metabolism, being a part of more than 300 enzymes, which are responsible for several biological processes. Its deficiency could result in enhanced oxidative stress, leading to damage in DNA, proteins, and lipids. It is suggested that inadequate serum levels of zinc could be correlated with cancer incidence, especially in the development and progression of prostate, pancreatic, and breast cancer [50]. In the population-based case-control study, Dimitropolou et al. [51] examined the relations between the dietary zinc intake and the incidence of brain tumors in adults. The authors collected the self-reported questionnaires of dietary food frequency from 637 brain cancer cases (of which 436 were gliomas) and 876 controls. In this study, the daily zinc intake ranged from 2.2 to 21.9 mg/day. In glioma patients, 30% had the zinc intake on the lowest quartile (Q1: 2.2–9.1 mg/day), while in the rest of the study group, 23% had the zinc intake from 9.2 to 10.3 mg/day (Q2), 22% consumed from 10.4 to 12.0 mg/day (Q3), and the rest (25%) were provided 12.1–21.9 mg/day (Q4) of this nutrient with a diet. In turn, no differences were found between individual levels of intake (Q1–Q4) in the control group. No associations were observed for the zinc intake and glioma incidence, even after adjustment of cofounders. No significant interactions were also observed, considering Ca, Fe, Cu, P, PUFA, protein, and fiber. The levels of consumption recorded were grouped by quartiles of the control distribution; the lowest category was used as a reference group. The significant relations were determined for Zn-disease and Fe associations (overall significance, *p* = 0.05) [51].

With regard to the studies on the effect of antioxidants on the cancer patients’ survival, the previous studies have shown divergent results since it has been demonstrated that antioxidants can increase the tumor resistance to chemotherapy or radiotherapy, which could contribute to the shorter survival time or antioxidants might reduce the toxicity of treatment in relations to healthy tissues, which on the other hand, would improve the patients’ survival [52,53]. Brain tissues are vulnerable to oxidative stress because of their low antioxidant defenses in the brain in comparison with other tissue. Additionally, antioxidants could prevent radiation damage [53]. Il’yasova et al. [54] assessed the total dietary antioxidant index and its relations to survival in 814 patients with glioblastoma from the San Francisco Bay Area Adult Glioma Study (SFBAAGS). Dietary antioxidant intake was collected by using the food frequency questionnaire and then was calculated according to the Trolox equivalent per gram. The researchers included patients in three series over the years 1991 and 2004, which is why there were differences in treatment methods used between the series I and II and the series III. The difference primarily referred to the fact that in the series III, during which 363 patients were included in the study, 41% of them received combination therapy (resection/radiation/temodar), which was not used in the previous series. The authors observed no relation between the survival and the dietary antioxidant index in the combined three series, even when age, gender, or race were taken into account. After considering vitamin supplements, no relationship was found, either. Only in the III series, investigated separately, there was a weak, though statistically relevant, the correlation between the higher antioxidant intake and the better survival with the tendency to be stronger on a log-scale (HR: 0.58, 95% CI: 0.46–0.74). However, after considering the treatment standard, this relationship was no longer significant. Although no significant relationship between dietary antioxidant intake and impact on the survival of GBM patients was demonstrated in Il’yasova et al., rapidly fatal GBM and the possible effect of food antioxidants on survival (III series) prompt an in-depth study on this topic [54].

In turn, Tedeschi-Blok et al. [55] studied the data from the same research as Il’yasova et al. (SFBAAGS). However, their analysis did not include the information from the series III. The questionnaires from 802 cancer patients and 846 controls were taken into consideration. The authors observed statistically relevant inverse association in the self-reported glioma cases (*n* = 497) compared to the controls in the case of the total antioxidant index (*p* < 0.05) and daidzein intake (*p* < 0.05) with a diet. While analyzing the data for all cases (*n* = 802), it was demonstrated that the total antioxidant index (*p* < 0.003), carotenoids (*p* < 0.05), and phytoestrogens—daidzein (*p* = 0.003), matairesinol (*p* < 0.05), secoisolariciresinol (*p* < 0.003), and cumasterol (*p* < 0.003)—were statistically significantly inversely associated with glioma risk [55].

Lycopene has been proven as an anti-invasion and pro-apoptotic compound, which could cross the blood-brain barrier [56]. Nutrition supplements have been indicated to be potentially useful as adjuvant therapy in high-grade gliomas. In the pilot study, among 50 high-grade glioma cases, patients were randomly divided into two groups: the first group received 8 mg oral lycopene daily with radiotherapy, and the second was given a placebo; in both groups, chemotherapy of paclitaxel was applied (patients were recruited for the study when temozolomide was not included in the standard treatment). The scientists measured pre- and post-radiotherapy plasma lycopene. The observation, as well as the clinical examination, was carried out for 3 months (Magnetic Resonance Imaging and Single Photon Emission Computed Tomography). At the baseline, lycopene levels did not differ between groups. After the radiotherapy cycle, there was an increase in the lycopene level in both groups (lycopene group: 152 ng/mL and 316 ng/mL; controls: 93 ng/mL and 98 ng/mL), while the levels were significantly higher in supplemented group than in the control group (*p* = 0.009). In the study group, tumor progression time was longer (40.83 vs. 26.74 weeks, *p* = 0.089), though not statistically relevant. Moreover, after completing the research, Puri et al. [57] found the statistically significantly higher median follow-up time in the group supplemented with lycopene (66.29 vs. 38.71 weeks, *p* = 0.05). It has been suggested that lycopene supplementation may be used as an adjunct to glioma treatment, but these findings require confirmation in the larger studies using the current treatment standard.

There is limited but positive evidence of the role of vitamin A and C in decreasing the glioma risk. This protective effect exists, especially among Americans, since most of the studies discussed in the meta-analyses above were carried out in the USA. Therefore, it seems rational to emphasize the role of proper nutrition in the prevention of gliomas, particularly the consumption of food products that provide adequate amounts of these antioxidants.

## 4. Lifestyle Factors

### 4.1. Anthropometric Indicators

Many dietary factors are related to the selected anthropometric indicators. Anthropometry is commonly used to measure the general nutritional status and predict health, performance, and survival of individuals. The indicators most frequently used are weight, height, and circumferences (waist, arm). Among determinants of adult’s height are genetic and environmental factors, of which the childhood nutritional status seems to play an important role. Higher levels of insulin-like growth factor (IGF) theoretically may be responsible for linking the growth achieved with the occurrence of individual cancers. Importantly, the height cannot be modifiable, in contrast to body weight. The results from the Million Women Study (MWS) showed that tallness could be seen as a potential risk factor for glioma development in adults; the risk of glioblastoma increased by about 20% for every additional 10 cm of growth [58]. Moore et al. [59] investigated the prospective data from The National Institutes of Health and the American Association of Retired Persons (NIH-AARP) Diet and Health Study and also observed the two-fold higher risk of glioma and glioblastoma in taller participants (>1.90 m or above) compared to shorter adults (<1.60 m; RR: 2.12, 95% CI: 1.18–3.81) [59]. Wiedmann et al. [60] found a similar relationship in the Norwegian population; every 10 cm higher height was associated with the increased risk of glioblastoma and other glioma types (HR for glioblastoma: 1.24, 95% CI: 1.17–1.31, HR for other gliomas: 1.18, 95% CI: 1.09–1.29) [60]. However, the statistical analysis performed by Cote et al. [61] using data from two prospective cohort studies—Nurses’ Health Study (NHS) and the Health Professionals Follow-Up Study (HPFS)—showed that height was associated with a higher incidence of glioma, solely among women (HR: 1.09, 95% CI: 1.04–1.14) [61]. On the other hand, the researchers of the large European cohort study (European Prospective Investigation into Cancer and Nutrition- EPIC) found no association between the height and glioma risk [62]. The meta-analysis of 13 prospective and two case-control studies carried out by Kitahara et al. [63] on 1354 glioma cases and the control group of 4734 subjects showed the borderline positive relationship between the height attained and the risk of glioma incidence (OR: 1.05, 95% CI: 0.99–1.13). Taking into account the gender division, no significant relationship was observed among women (cancer cases: 593, controls: 1587). However, a positive relationship was noted after separating individual growth intervals among men (cancer cases: 761, controls: 3147). Glioma risk was higher in the individuals with the height equal or above 1.90 m compared to the participants with the height in 1.70–1.75 m range (OR: 1.70, 95% CI: 1.11–2.61). Narrowing down the criteria only to glioblastoma, the relationship seemed to be clearer; the risk of glioblastoma increased more in the taller men (OR: 1.99) than in the shorter men (OR: 1.35). Similarly, for women, a height in the range of 1.70–1.74 m was associated with the increased risk of glioma compared to a height in the range of 1.60–1.64 m (OR: 1.44, 95% CI: 1.05–1.98). However, as in men, the risk was higher after specifying the glioblastoma group (OR for 1.70–1.74 m: 1.87). Interestingly, in women, unlike men, the risk of glioma in the higher height decreased (OR: 1.06) [63]. Although there are scientific reports about the relationship between the height attained and the risk of glioma and glioblastoma, there is still a lack of explanatory evidence of biological mechanisms that contribute to these relationships.

Excessive body weight is considered a risk factor not only for the development of several types of cancer but also for the survival and prognosis of cancer patients. Statements of two organizations—IARC and WCRF (World Cancer Research Found)—are consistent and indicate that overweight or obesity (the body mass index-BMI 25–29.9 kg/m^2^ or over 30 kg/m^2^) is one of the causes of several cancers: colon and rectum, endometrium, ovary, esophagus, gallbladder, pancreas, liver, prostate (advanced), thyroid, multiple myeloma, and renal cancer [7,64]. Many potential mechanisms of excessive body weight have been described in particular cancers so far, but they are not entirely clear. Therefore, it seems important to maintain body weight within the normal range to reduce the risk of specific types of cancer [64,65].

It must be highlighted that obesity was the strongest lifestyle factor in NIH-AARP Diet and Health study, enhancing the glioma risk to the greatest extend. Adults, who were obese at the age of 18, had nearly 4 times increased glioma risk in comparison to participants with normal weight (RR: 3.74, 95% CI: 2.03–6.90) [60]. Similar results have been reported in NHS and HPFS studies; BMI greater than or equal 25 kg/m^2^ at the young age (18 years in NHS and 21 in HPFS) was related to a slightly elevated glioma risk (pooled cohorts HR: 1.35, 95% CI: 1.06–1.72), whereas adult BMI and circumference of waist had no significant association with glioma incidence. Furthermore, the researchers determine no pre-diagnostic changes in body weight for 8 years before diagnosis [62]. Excessive body weight and disproportionate BMI (too high) were risk factors for glioma development in Dai et al.’s [66] meta-analysis, which involved 3726 cases based on five articles with six studies. Overweight (pooled HR: 1.12, 95% CI: 1.02–1.22) and obesity (pooled HR: 1.14, 95% CI: 1.02–1.27) were related to higher glioma incidence, while underweight (pooled HR: 1.08, 95% CI: 0.74–1.58) was associated with the decreased risk [66]. However, among Norwegian men and women, no relationship was found between excessive body weight and glioma risk [61]. The meta-analysis of 12 studies on BMI and the risk of glioma and meningioma proved that overweight (RR: 1.21, 95% CI: 1.01–1.43) and obesity (RR: 1.54, 95% CI: 1.32–1.79) were related to the enhanced risk of meningioma but not glioma (overweight RR: 1.06, 95% CI: 0.95–1.20, obesity RR: 1.11, 95% CI: 0.98–1.27) [67]. On the other hand, underweight (BMI < 18.5 kg/m^2^) at the age of 21 in the study conducted by Little et al. [68] was associated with a lower risk of glioma in females [68]. Sergentanis et al. [69] meta-analyzed, among others, the relationship between the excessive body weight (overweight and obesity) and the risk of glioma based on six studies among women (four cohorts and two case-control studies) and six among men (two cohorts and four case-control studies). In addition, they investigated the studies without gender division (11 cohort and three case-control studies). Excessive body weight (BMI ≥ 25 kg/m^2^) in women increased the risk of glioma (RR: 1.17, 95% CI: 1.03–1.32), and a statistically relevant relationship was reported in the group of overweight females (RR: 1.19, 95% CI: 1.02–1.38), while in the case of obese women, there was no such relationship. In the studies on men and the studies not taking into account the gender division, no significant relationship was found between overweight, obesity, or overweight and obesity combined and the risk of developing glioma [69].

Kabat et al. [70], in turn, investigated the data collected in Women’s Health Initiative (WHI) study, which was based on the clinical and observational study (clinical trial, *n* = 68,132; observational study, *n* = 93,676), and compared the relationship between the anthropometric indicators and the risk of glioma in postmenopausal women aged 50–79. At the time of entering the study, all participants were subjected to anthropometric measurements carried out by the trained staff, while the data on anthropometric indicators from the years preceding the study were taken solely from the observational study (*n* = 93,676). In Kabat et al.’s study [70], there were 217 glioma cases, of which 167 were glioblastoma, and the follow-up time was 18 years. The results showed a modest linear trend of glioma and glioblastoma risk with increasing BMI and the waist-hip ratio (WHR), but no association was observed for the waist circumference. In addition, the information on body weight changes over time (18, 35, and 50 years old), collected for the group included in the observational study, showed no significant relationship, either [70].

Hence, based on the results above, the time of occurrence of excessive body weight seems to be important. Being overweight or obese before the age of 18 (or 21 years in some analysis) may increase the risk of developing glioblastoma, while the excessive body weight achieved in adulthood seems to have a little impact.

### 4.2. Physical Activity

The levels of physical activity for recommended adults (18–64) to maintain health include undertaking at least 150 min of moderate-intensity aerobic activity or 75 min of vigorous-intensity aerobic activity per week. Increasing the time of types of physical activity mentioned above could additionally be beneficial. It is also suggested to include strength training twice a week involving major muscle groups [7]. There is strong evidence that adequate levels of physical activity reduce the risk of colon, breast (postmenopausal), and endometrial cancer development. It is speculated that the beneficial effects of physical activity in preventing specific types of cancer are based on reducing insulin resistance and inflammation. Additionally, physical activity affects many metabolic, immunologic, and hormonal (including IGF-1) pathways [7]. Elevated serum IGF-1 levels have been related to a higher risk of glioma [71]. The importance of undertaking physical activity during adolescence is also crucial in glioma prevention—the survey participants of NIH-AARP Diet and Health Study in the 15–18 age group had a 36% lower risk compared to inactive people [60]. In Niedermaier et al.’s [67] study, which included a comparison of six studies, it was demonstrated that the adequate physical activity level was weakly inversely correlated with the risk of glioma incidence [67].

Although we have no influence on height attained, it seems important to pay attention to maintaining proper body weight, especially in adolescence, to reduce the glioma risk. For this reason, taking an appropriate level of daily physical activity could also be beneficial in the prevention of glioma development.

A summary of the aspects reviewed in this paper is presented in Table 1.

## 5. Summary

Considering the facts presented above, it is difficult to associate nutritional and lifestyle factors with the incidence of gliomas due to their complex etiology. Dietary factors that may significantly affect the risk of developing glioma are still not clearly identified and described. Based on the available literature, it can be assumed that coffee and tea consumption and the intake of adequate vegetables and antioxidants (such as vitamins C and A) could have a protective effect. While other factors like alcohol intake, red meat, and red processed meat consumption, as well as iron intake (both heme and non-heme), have shown no correlation with the incidence of glioma. In turn, the consumption of fish, coffee, and tea does not increase the risk, but further studies are necessary to determine whether they may prevent glioma. Maintaining a proper weight of the body, as well as undertaking a sufficient level of daily physical activity, also seems to be important, especially during adolescence. Higher BMI has increased the risk of glioma. The attained height, which cannot be modifiable in contrast to body weight, has shown a positive association with glioma development. However, there is still an insufficient number of studies in the field of nutritional factors and the risk of glioma. In order to link more accurately selected nutritional factors to the incidence of gliomas, it seems necessary to conduct large, prospective, controlled studies in the different world regions.

## Figures and Tables

**Table 1:** The dietary and lifestyle factors discussed in relation to glioma incidence.

Dietary/Lifestyle Factor	Effect on Glioma Incidence	Based on Evidence [Ref.]
Alcohol	N/I *	Meta-analysis, *n* = 1,462,336, GC ** = 4247
		(19 studies: 4 cohort and 15 case-control) [11]
		Case-control study, *n* = 1479, Neuroepithelial tumors cases = 494, of which GC = 103 [12]
		Combined 3 cohort studies, *n* = 1,262,104, GC = 2313 [13]
Coffee	Protective	Meta-analysis, *n* = 1,684,262, GC = 2583
		(11 studies: 8 cohort and 3 case-control) [20]
	N/I	Meta-analysis, *n* = 1,320,889, GC = 2075
		(6 studies: 4 cohort and 2 case-control) [19]
		Combined 3 cohort studies, *n* = 237,516, GC = 554 [21]
		Combined 3 cohort studies, *n* = 1,262,104, GC = 2313 [13]
Tea	Protective	Meta-analysis, *n* = 1,186,950, GC = 1797
		(4 studies: 3 cohort and 1 case-control) [19]
		Combined 3 cohort studies,
		*n* = 237,516, GC = 554 [21]
	N/I	Combined 3 cohort studies, *n* = 1,262,104, GC = 2313 [13]
		Meta-analysis, *n* = 1,510,397, *n* = 2226
		(8 studies: 6 cohort and 2 case-control) [20]
Both Coffee and Tea	Protective	Meta-analysis, *n* = 864,387, GC = 1582
		(3 cohort studies) [19]
Fruit	Protective in Asians	Meta-analysis, *n* = N/A ***, GC = 377
		(5 case-control studies:) [24]
	N/I	Case-control study, *n* = 1479, Neuroepithelial tumors cases = 494, of which GC = 103 [12]
		Meta-analysis, *n* = N/A, GC = 3994
		(17 studies: 15 case-control, 2 prospective) [24]
Vegetables	Protective (vegetables)	Meta-analysis, *n* = N/A, GC = 5562
		(15 studies: 14 case-control, 1 prospective) [24]
	Protective (yellow-orange and the green-leafy)	Case-control study, *n* = 4034, GC = 1185 [26]
	N/I	Case-control study, *n* = 1479, Neuroepithelial tumors cases = 494, of which GC = 103 [12]
Fish	Protective	Case-control study, *n* = 348, GC = 128 [30]
	N/I	Combined 3 cohort studies, *n* = 1,262,104, GC = 2313 [13]
		Meta-analysis, *n* = 501,617, Brain tumor cases = 4428, GC = 854
		(5 case-control studies) [29]
Red Meat	Increasing the risk	Meta-analysis, *n* = 37,802, GC = 2181
		(6 studies: 1 cohort, 5 case-control) [35]
	N/I	Meta-analysis, *n* = 836,370, GC = 1156,
		(3 studies: 2 cohort, 1 case-control) [34]
		Cohort study, *n* = 408,751, GC = 688 [36]
Processed Meat	Increasing the risk	Meta-analysis, *n* = 877,640, GC = 3896,
		(14 studies: 3 prospective, 11 case-control) [34]
		Meta-analysis, *n* = 820,660, GC = 5058
		(17 studies: 3 cohort, 14 case-control) [35]
	N/I	Cohort study, *n* = 408,751, GC = 688 [36]
Total (red meat and red processed meat)	N/I	Meta-analysis, *n* = 781,707, GC = 2172
		(4 studies: 2 cohort, 2 case-control) [35]
Nitrate	N/I	Meta-analysis, *n* = N/A, GC = 1771
		(7 studies: 2 cohort, 5 case-control) [37]
		Meta-analysis, *n* = N/A, GC = N/A,
		(5 studies: 2 cohort, 3 case-control) [38]
Nitrite	Increasing the risk	Meta-analysis, *n* = N/A, GC = N/A,
		(6 studies: 2 cohort, 4 case-control) [38]
	N/I	Meta-analysis, *n* = N/A, GC = 2264
		(9 studies: 2 cohort, 7 case-control) [37]
Vitamin E	N/I	Meta-analysis, *n* = N/A, GC = 3180
		(10 studies: 2 cohort, 8 case-control) [42]
Vitamin A	Protective	Meta-analysis, *n* = 4123, GC = 1841
		(7 case-control studies) [46]
Vitamin C	Protective among Americans	Meta-analysis, *n* = N/A, GC = 3409 (15 studies: 2 cohort, 13 case-control) [49]
Zinc	N/I	Case-control study, *n* = 1513, GC = 637 [51]
Height	Increasing the risk	Cohort study, *n* = 1,300,000, GC = 646 [58]
		Cohort study *n* = 270,395, GC = 480 [59]
		Cohort study *n* = 1,800,000, GC = 4382 [60]
		2 cohort studies *n* = 173,096, GC = 508 [61]
		Meta-analysis, *n* = 6088, GC = 1354
		(15 studies: 13 prospective, 2 case-control) [63]
	N/I	Cohort study, *n* = 380,775, GC = 340 [62]
Body Weight, BMI	Increasing the risk	Cohort study *n* = 1,800,000, GC = 4382 [60]
		Cohort study, *n* = 380,775, GC = 340 [62]
		Meta-analysis, *n* = N/A, GC = 3762 (6 studies: 5 cohort, 1 case-control) [66]
		Meta-analysis, *n* = N/A, GC = 3057 (12 studies) [67]
		Meta-analysis, *n* = 10,156,370, GC = 3683
		(22 studies: 14 cohort, 8 case-control)
		[69]
		Cohort study, *n* = 92,557, GC = 217 [70]
	N/I	2 cohort studies, *n* = 173,096, GC = 508 [61]
Physical Activity	Protective	Cohort study *n* = 1,800,000, GC = 4382 [60]
		Meta-analysis, *n* = N/A, GC = 3057
		(12 studies) [67]

* N/I-no influence, ** GC-glioma cases, *** N/A-not available.

## References

[1] Bray F., Ferlay J., Soerjomataram I., Siegel R.L., Torre L.A., Jemal A. (2018). Global cancer statistics 2018: Estimates of Incidence and Mortality Worldwide for 36 Cancers in 185 Countries. Ca. Cancer J. Clin..

[2] Christ A., Lauterbach M., Latz E. (2019). Western Diet and the Immune System: An Inflammatory Connection. Immunity.

[3] Goodenberger M.L., Jenkins R.B. (2012). Genetics of adult glioma. Cancer Genet..

[4] Molinaro A.M., Taylor J.W., Wiencke J.K., Wrensch M.R. (2019). Genetic and molecular epidemiology of adult diffuse glioma. Nat. Rev. Neurol..

[5] Pisapia D.J. (2017). The Updated World Health Organization Glioma Classification: Cellular and Molecular Origins of Adult Infiltrating Gliomas. Arch. Pathol. Lab. Med..

[6] Weller M., van den Bent M., Tonn J.C., Stupp R., Preusser M., Cohen-Jonathan-Moyal E., Henriksson R., Le Rhun E., Balana C., Chinot O. (2017). European Association for Neuro-Oncology (EANO) guideline on the diagnosis and treatment of adult astrocytic and oligodendroglial gliomas. Lancet Oncol..

[7] World Cancer Research Found/American Institute for Cancer Research Diet, nutrition, physical activity and cancer: a global perspective. Continuous update project expert report. http://dietandcancerreport.org.

[8] Harper C. (1998). The neuropathology of alcohol-specific brain damage, or does alcohol damage the brain?. J. Neuropathol. Exp. Neurol..

[9] Cogliano V.J., Baan R., Straif K., Grosse Y., Lauby-Secretan B., El Ghissassi F., Bouvard V., Benbrahim-Tallaa L., Guha N., Freeman C. (2011). Preventable exposures associated with human cancers. J. Natl. Cancer Inst..

[10] Schwedhelm C., Boeing H., Hoffmann G., Aleksandrova K., Schwingshackl L. (2016). Effect of diet on mortality and cancer recurrence among cancer survivors: a systematic review and meta-analysis of cohort studies. Nutr. Rev..

[11] Qi Z.Y., Shao C., Yang C., Wang Z., Hui G.Z. (2014). Alcohol consumption and risk of glioma: a meta-analysis of 19 observational studies. Nutrients.

[12] Allès B., Pouchieu C., Gruber A., Lebailly P., Loiseau H., Fabbro-Peray P., Letenneur L., Baldi I. (2016). Dietary and alcohol intake and central nervous system tumors in adults: results of the CERENAT multicenter case-control study. Neuroepidemiology.

[13] Kuan A.S., Green J., Kitahara C.M., Key T., Reeves G.K., Floud S., Balkwill A., Bradbury K., Liao L.M., Freedman N.D. (2019). Diet and risk of glioma: combined analysis of 3 large prospective studies in the UK and USA. Neuro Oncol..

[14] Ratna A., Mandrekar P. (2017). Alcohol and Cancer: Mechanisms and Therapies. Biomolecules.

[15] Poole R., Kennedy O.J., Roderick P., Fallowfield J.A., Hayes P.C., Parkes J. (2017). Coffee consumption and health: umbrella review of meta-analyses of multiple health outcomes. BMJ.

[16] Huber W.W., Scharf G., Nagel G., Prustomersky S., Schulte-Hermann R., Kaina B. (2003). Coffee and its chemopreventive components Kahweol and Cafestol increase the activity of O6-methylguanine-DNA methyltransferase in rat liver–comparison with phase II xenobiotic metabolism. Mutat Res..

[17] Shirakami Y., Shimizu M. (2018). Possible mechanisms of green tea and its constituents against cancer. Molecules.

[18] Le C.T., Leenders W., Molenaar R., Cornelis N. (2018). Effects of the green tea polyphenol epigallocatechin-3-gallate on glioma: a critical evaluation of the literature. Nutr. Cancer.

[19] Malerba S., Galeone C., Pelucchi C., Turati F., Hashibe M., La Vecchia C., Tavani A. (2013). A meta-analysis of coffee and tea consumption and the risk of glioma in adults. Cancer Causes Control.

[20] Song Y., Wang Z., Jin Y., Guo J. (2019). Association between tea and coffee consumption and brain cancer risk: an updated meta-analysis. World J. Surg. Oncol..

[21] Cote D.J., Bever A.M., Wilson K.M., Smith T.R., Smith-Warner S.A., Stampfer M.J. (2020). A prospective study of tea and coffee intake and risk of glioma. Int. J. Cancer.

[22] Kang S.S., Han K.S., Ku B.M., Lee Y.K., Hong J., Shin H.Y., Almonte A.G., Woo D.H., Brat D.J., Hwang E.M. (2010). Caffeine-mediated inhibition of calcium release channel inositol 1,4,5-trisphosphate receptor subtype 3 blocks glioblastoma invasion and extends survival. Cancer Res..

[23] Aune D., Giovannucci E., Boffetta P., Fadnes L.T., Keum N., Norat T., Greenwood D.C., Riboli E., Vatten L.J., Tonstad S. (2017). Fruit and vegetable intake and the risk of cardiovascular disease, total cancer and all-cause mortality-a systematic review and dose-response meta-analysis of prospective studies. Int. J. Epidemiol..

[24] Ying B.S. (2014). Association between fruit and vegetable intake and risk for glioma: a meta-analysis. Nutrition.

[25] Holick C.N., Giovannucci E.L., Rosner B., Stampfer M.J., Michaud D.S. (2007). Prospective study of intake of fruit, vegetables, and carotenoids and the risk of adult glioma. Am. J. Clin. Nutr..

[26] Terry M.B., Howe G., Pogoda J.M., Zhang F.F., Ahlbom A., Choi B., Giles G.G., Little J., Lubin F., Menegoz F. (2009). An international case-control study of adult diet and brain tumor risk: A histology-specific analysis by food group. Ann. Epidemiol..

[27] McCullough M.L., Giovannucci E.L. (2004). Diet and cancer prevention. Oncogene.

[28] Siriwardhana N., Kalupahana N.S., Moustaid-Moussa N. (2012). Health benefits of n-3 polyunsaturated fatty acids: eicosapentaenoic acid and docosahexaenoic acid. Adv. Food Nutr. Res..

[29] Lian W., Wang R., Xing B., Yao Y. (2017). Fish intake and the risk of brain tumor: A meta-analysis with systematic review. Nutr. J..

[30] Dadfarma A., Shayanfar M., Benisi-Kohansal S., Mohammad-Shirazi M., Sharifi G., Hosseini G., Esmaillzadeh A. (2018). Dietary polyunsaturated fat intake in relation to glioma: a case-control study. Nutr. Cancer..

[31] Serini S., Calviello G. (2018). Long-chain omega-3 fatty acids and cancer: any cause for concern?. Curr. Opin. Clin. Nutr. Metab. Care..

[32] Tricker A.R. (1997). N-nitroso compounds and man: sources of exposure, endogenous formation and occurrence in body fluids. Eur. J. Cancer Prev..

[33] Nagao M., Tsugane S. (2016). Cancer in Japan: Prevalence, prevention and the role of heterocyclic amines in human carcinogenesis. Genes Environ..

[34] Wei Y., Zou D., Cao D., Xie P. (2015). Association between processed meat and red meat consumption and risk for glioma: a meta-analysis from 14 articles. Nutrition.

[35] Saneei P., Willett W., Esmaillzadeh A. (2015). Red and processed meat consumption and risk of glioma in adults: A systematic review and meta-analysis of observational studies. J. Res. Med. Sci..

[36] Ward H.A., Gayle A., Jakszyn P., Merritt M., Melin B., Freisling H., Weiderpass E., Tjonneland A., Olsen A., Dahm C.C. (2018). Meat and heme iron intake in relation to glioma in the European Prospective Investigation into Cancer and Nutrition study. Eur. J. Cancer Prev..

[37] Wang P., Chong-Xian H., Yuanyuan L., Dong Z. (2016). Dietary nitrite and nitrate is not associated with adult glioma risk: a meta-analysis. Int. J. Clin. Exp. Med..

[38] Xie L., Mo M., Jia H.X., Liang F., Yuan J., Zhu J. (2016). Association between dietary nitrate and nitrite intake and sitespecific cancer risk: evidence from observational studies. Oncotarget.

[39] Mut-Salud N., Álvarez P.J., Garrido J.M., Carrasco E., Aránega A., Rodríguez-Serrano F. (2016). Antioxidant intake and antitumor therapy: toward nutritional recommendations for optimal results. Oxid. Med. Cell Longev..

[40] Das Gupta S., Suh N. (2016). Tocopherols in cancer: An update. Mol. Nutr. Food Res..

[41] Jain A., Tiwari A., Verma A., Jain S.K. (2017). Vitamins for Cancer Prevention and Treatment: An Insight. Curr. Mol. Med..

[42] Qin S., Wang M., Zhang T., Zhang S. (2014). Vitamin E intake is not associated with glioma risk: evidence from a meta-analysis. Neuroepidemiology.

[43] Dubrow R., Darefsky A.S., Park Y., Mayne S.T., Moore S.C., Kilfoy B., Cross A.J., Sinha R., Hollenbeck A.R., Schatzkin A. (2010). Dietary components related to N-nitroso compound formation: a prospective study of adult glioma. Cancer Epidemiol. Biomarkers Prev..

[44] Liang C., Yang L., Guo S. (2015). All-trans retinoic acid inhibits migration, invasion and proliferation, and promotes apoptosis in glioma cells in vitro. Oncol. Lett..

[45] Bouterfa H., Picht T., Kess D., Herbold C., Noll E., Black P.M., Roosen K., Tonn J.C. (2000). Retinoids inhibit human glioma cell proliferation and migration in primary cell cultures but not in established cell lines. Neurosurgery.

[46] Lv W., Zhong X., Xu L., Han W. (2015). Association between dietary vitamin A intake and the risk od glioma: evidence from a meta-analysis. Nutrients.

[47] DeLorenze G.N., McCoy L., Tsai A.L., Quesenberry C.P., Rice T., Il’yasova D., Wrensch M. (2010). Daily intake of antioxidants in relation to survival among adult patients diagnosed with malignant glioma. BMC Cancer.

[48] Naidu K.A., Tang J.L., Naidu K.A., Prockop L.D., Nicosia S.V., Coppola D. (2001). Antiproliferative and apoptotic effect of ascorbyl stearate in human glioblastoma multiforme cells: modulation of insulin-like growth factor-I receptor (IGF-IR) expression. J. Neurooncol..

[49] Zhou S., Wang X., Tan Y., Qiu L., Fang H., Li W. (2015). Association between vitamin C intake and glioma risk: evidence from a meta-analysis. Neuroepidemiology.

[50] Hoang B.X., Han B., Shaw D.G., Nimni M. (2016). Zinc as a possible preventive and therapeutic agent in pancreatic, prostate, and breast cancer. Eur. J. Cancer Prev..

[51] Dimitropoulou P., Nayee S., Liu J.F., van Tongeren M., Hepworth S.J., Muir K.R. (2008). Dietary zinc intake and brain cancer in adults: a case-control study. Br. J. Nutr..

[52] Lawenda B.D., Kelly K.M., Ladas E.J., Sagar S.M., Vickers A., Blumberg J.B. (2008). Should supplemental antioxidant administration be avoided during chemotherapy and radiation therapy?. J. Natl. Cancer. Inst..

[53] Conklin K.A. (2004). Chemotherapy-associated oxidative stress: impact on chemotherapeutic effectiveness. Integr. Cancer Therapies.

[54] Il’yasowa D., Marcello J.E., McCoy L., Rice T., Wrensch M. (2009). Total dietary antioxidant index and survival in patients with glioblastoma multiforme. Cancer Causes Control.

[55] Tedeschi-Blok N., Lee M., Sison J.D., Miike R., Wrensch M. (2006). Inverse association of antioxidant and phytoestrogen nutrient intake with adult glioma in the San Francisco Bay Area: a case-control study. BMC Cancer.

[56] Rooprai H.K., Christidou M., Pilkington G.J. (2003). The potential for strategies using micronutrients and heterocyclic drugs to treat invasive gliomas. Acta Neurochir..

[57] Puri T., Goyal S., Julka P.K., Nair O., Sharma D.N., Rath G.K. (2010). Lycopene in treatment of high-grade gliomas: A pilot study. Neurol India.

[58] Benson V.S., Pirie K., Green J., Casabonne D., Beral V. (2008). Lifestyle factors and primary glioma and meningioma tumors in the Million Women Study cohort. Br. J. Cancer..

[59] Moore S.C., Rajaraman P., Dubrow R., Darefsky A.S., Koebnick C., Hollenbeck A., Schatzkin A., Leitzmann M.F. (2009). Height, body mass index, and physical activity in relations to glioma risk. Cancer. Res..

[60] Wiedmann M.K.H., Brunborg C., Di Ieva A., Lindemann K., Johannesen T.B., Vatten L., Helseth E., Zwart J.A. (2017). The impact of body mass index and height on the risk for glioblastoma and other glioma subgroups: a large prospective cohort study. Neuro Oncol..

[61] Cote D.J., Downer M.K., Smith T.R., Smith-Warner S.A., Egan K.M., Stampfer M.J. (2018). Height, waist circumference, body mass index, and body somatotype across the life course and risk of glioma. Cancer Causes Control..

[62] Michaud D.S., Bové G., Gallo V., Schlehofer B., Tjønneland A., Olsen A., Overvad K., Dahm C.C., Teucher B., Boeing H. (2011). Anthropometric measures, physical activity, and risk of glioma and meningioma in a large prospective cohort study. Cancer Prev. Res..

[63] Kitahara C.M., Wang S.S., Melin B.S., Wang Z., Braganza M., Inskip P.D., Albanes D., Andersson U., Freeman L.E.B., Buring J.E. (2012). Association between adult height, genetic susceptibility and risk of glioma. Int. J. Epidemiol..

[64] Lauby-Secretan B., Scoccianti C., Loomis D., Grosse Y., Bianchini F., Straif K. (2016). International Agency for Research on Cancer Handbook Working Group. Body Fatness and Cancer-viewpoint of the IARC Working Group. N. Engl. J. Med.

[65] Colditz G.A., Peterson L.L. (2018). Obesity and cancer: evidence, impact, and future directions. Clin. Chem..

[66] Dai Z., Huang Q., Liu H. (2015). Different body mass index grade on the risk of developing glioma: a meta-analysis. Chin. Neurosurg. J..

[67] Niedermaier T., Behrens G., Schmid D., Schlecht I., Fischer B., Leitzmann M.F. (2015). Body mass index, physical activity, and risk of adult meningioma and glioma: A meta-analysis. Neurology.

[68] Little R.B., Madden M.H., Thompson R.C., Olson J.J., LaRocca R.V., Pan E., Browning J.E., Egan K.E., Nabors L.B. (2013). Anthropometric factors in relation to risk of glioma. Cancer Causes Control..

[69] Sergentanis T.N., Tsivgoulis G., Perlepe C., Stathopoulos I.N., Tzanninis I.G., Sergentanis I.N., Psaltopoulou T. (2015). Obesity and risk for brain/CNS tumors, gliomas and meningiomas: a meta-analysis. PLoS ONE.

[70] Kabat G.C., Rohan T.E. (2018). Adiposity at different periods of life and risk of adult glioma in a cohort of postmenopausal women. Cancer Epidemiol..

[71] Lönn S., Inskip P.D., Pollak M.N., Weinstein S.J., Virtamo J., Albanes D. (2007). Glioma risk in relation to serum levels of insulin-like growth factors. Cancer Epidemiol. Biomarkers Prev..

